# Evaluating Interlaboratory Variability in Wastewater-Based COVID-19 Surveillance

**DOI:** 10.3390/microorganisms13030526

**Published:** 2025-02-27

**Authors:** Arianna Azzellino, Laura Pellegrinelli, Ramon Pedrini, Andrea Turolla, Barbara Bertasi, Sandro Binda, Sara Castiglioni, Clementina E. Cocuzza, Fabio Ferrari, Andrea Franzetti, Maria Giovanna Guiso, Marina Nadia Losio, Marianna Martinelli, Antonino Martines, Rosario Musumeci, Desdemona Oliva, Laura Sandri, Valeria Primache, Francesco Righi, Annalisa Scarazzato, Silvia Schiarea, Elena Pariani, Emanuela Ammoni, Danilo Cereda, Francesca Malpei

**Affiliations:** 1 Department of Civil and Environmental Engineering, Politecnico di Milano, 20133 Milan, Italy; ramon.pedrini@polimi.it (R.P.); andrea.turolla@polimi.it (A.T.); francesca.malpei@polimi.it (F.M.); 2 Department of Biomedical Sciences of Health, University of Milan, 20133 Milan, Italy; laura.pellegrinelli@unimi.it (L.P.); sandro.binda@unimi.it (S.B.); laura.sandri@unimi.it (L.S.); valeria.primache@unimi.it (V.P.); elena.pariani@unimi.it (E.P.); 3 Istituto Zooprofilattico Sperimentale della Lombardia e dell’Emilia-Romagna “B. Ubertini”, 25124 Brescia, Italy; barbara.bertasi@izsler.it (B.B.); marinanadia.losio@izsler.it (M.N.L.); francesco.righi@izsler.it (F.R.); annalisa.scarazzato@izsler.it (A.S.); 4 Department of Environmental Sciences, Istituto di Ricerche Farmacologiche Mario Negri IRCCS, 20156 Milan, Italy; sara.castiglioni@marionegri.it (S.C.); silvia.schiarea@marionegri.it (S.S.); 5 Department of Medicine and Surgery, University of Milano-Bicocca, 20900 Monza, Italy; clementina.cocuzza@unimib.it (C.E.C.); marianna.martinelli@unimib.it (M.M.); rosario.musumeci@unimib.it (R.M.); 6 CAP Holding Spa, 20142 Milan, Italy; fabio.ferrari@gruppocap.it (F.F.); mariagiovanna.guiso@gruppocap.it (M.G.G.); antonino.martines@gruppocap.it (A.M.); desdemona.oliva@gruppocap.it (D.O.); 7 Department of Earth and Environmental, Sciences—DISAT, University of Milano-Bicocca, 20126 Milan, Italy; 8 DG Welfare, Regione Lombardia, 20124 Milan, Italy; emanuela_ammoni@regione.lombardia.it (E.A.); danilo_cereda@regione.lombardia.it (D.C.)

**Keywords:** wastewater environmental surveillance, SARS-CoV2, interlaboratory ring test, detection methods, generalized linear models

## Abstract

Wastewater-based environmental surveillance enables the monitoring of SARS-CoV-2 dynamics within populations, offering critical epidemiological insights. Numerous workflows for tracking SARS-CoV-2 have been developed globally, underscoring the need for interlaboratory comparisons to ensure data consistency and comparability. An inter-calibration test was conducted among laboratories within the network monitoring SARS-CoV-2 in wastewater samples across the Lombardy region (Italy). The test aimed to evaluate data reliability and identify potential sources of variability using robust statistical approaches. Three wastewater samples were analyzed in parallel by four laboratories using identical pre-analytical (PEG-8000-based centrifugation) and analytical processes (qPCR targeting N1/N3 and Orf-1ab). A two-way ANOVA framework within Generalized Linear Models was applied, and multiple pairwise comparisons among laboratories were performed using the Bonferroni post hoc test. The statistical analysis revealed that the primary source of variability in the results was associated with the analytical phase. This variability was likely influenced by differences in the standard curves used by the laboratories to quantify SARS-CoV-2 concentrations, as well as the size of the wastewater treatment plants. The findings of this study highlight the importance of interlaboratory testing in verifying the consistency of analytical determinations and in identifying the key sources of variation.

## 1. Introduction

Wastewater environmental surveillance (WES) has been utilized for decades for public health monitoring and disease prevention. This approach involves the systematic collection and analysis of wastewater and other environmental samples to detect the presence of pathogens, toxins, and other hazardous substances. One prominent example of its application is the World Health Organization’s (WHO) recommendation in 2014 to use wastewater surveillance for poliovirus detection. Public health authorities can monitor poliovirus circulation in a community by analyzing sewage samples, even in the absence of clinical cases. This method allows for the early detection of outbreaks, facilitating timely interventions and vaccination campaigns to prevent the spread of the virus. Beyond poliovirus, WES has been employed to track a variety of infectious diseases, including poliovirus, hepatitis A, norovirus, and, more recently, SARS-CoV-2, the virus responsible for COVID-19 [1]. Since its emergence in 2020, COVID-19 has remained a major global public health concern, posing significant challenges to the scientific community, the general public, and society at large. The virus and its variants have caused considerable morbidity and mortality. By February 2025, five years after the emergence of SARS-CoV-2, the WHO reported approximately 777 million COVID-19 cases and over 7 million related deaths [2]. In response, unprecedented control measures have been implemented, including the administration of more than 13 billion vaccine doses and the adoption of enhanced surveillance activities, which include novel monitoring strategies.

WES may fill the gaps and overcome the limitations of clinical surveillance, such as a lack of access to healthcare samples, direct patient involvement, and cost. In August 2020, the WHO recognized the value of WES as a complementary approach for supporting COVID-19 clinical surveillance, and since then, significant progress has been made. During periods of low test willingness or low availability of clinical tests, WES has proven to be a valuable indicator tool to estimate the dynamics of the pandemic’s progression and individual testing strategies [3,4] to monitor the effectiveness of interventions ordered by local public health authorities during the COVID-19 pandemic [5,6]. WES saw rapid and substantial advancements globally, with multiple jurisdictions collaborating to broaden its scope, increase its reliability, and integrate it into routine public health surveillance. There is now a global interest in expanding WES for monitoring pathogens beyond SARS-CoV-2 [4]. A broad range of published in-house RNA isolation methods and commercially available complete workflows are today available for the extraction of nucleic acids from wastewater samples [7,8,9], with kits which generally contain reagents for the lysis of viruses and other particles in wastewater, followed by the binding of RNA to a column or magnetic beads and the subsequent washing and elution of the RNA. The purified RNA can then be used for various applications, such as SARS-CoV-2-specific RT-PCR. Regardless of whether commercial or publicly available methods are used, accurate and consistent analytical results are critical. To verify the consistency of the results and evaluate the accuracy of the analytical method, interlaboratory comparisons need to be routinely performed, in which the same blinded test samples are tested in several independent laboratories (proficiency testing). These interlaboratory comparisons may enable the identification of discrepancies and errors in the analytical results due to various factors and different pre-analytical and analytical steps such as sample concentration, acid nucleic extraction methods, and molecular assays carried out from each laboratory. However, according to a recent survey [10], very few laboratories involved with WES engage in proficiency testing exercises for SARS-CoV-2 surveillance, and neither analysis of these types of samples is included in the international “Quality Control for Molecular Diagnostics” (QCMD) program so far. In this regard, it is worth noting that QCMD is typically designed for clinical samples, and no QCMD is currently available for environmental samples.

Moreover, despite the extensive research efforts concerning the monitoring of SARS-CoV-2 in wastewater systems over the last few years, there is still a remarkable lack of standardized methods for the concentration, extraction, and quantification of viral RNA [10] and interpretation of the data, which complicates the comparison of data between laboratories [11,12]. In the few studies presenting interlaboratory comparisons [11,12,13], the lack of a uniform approach, both in the study design and in the approach of data analysis, has led to a variation in the results with no solid explanation for the observed discrepancies. This variability is also reflected in the differences observed in detection limits, which are influenced by the specific Standard Operating Procedures (SOPs) adopted by each laboratory. Pecson and colleagues [12] have also calculated the theoretical LOD of the SOPs in their study, which spanned seven orders of magnitude (i.e., the 10th and 90th percentiles of theoretical LOD spanned from 3.0- to 6.1 log gc/L).

The results of surveys conducted by Paracchini and colleagues [10] confirm this variability on a large scale, taking into account not only academia and industry but also the different methodologies used by national public health laboratories.

In the context of the WES program developed in the Lombardy region (with a population of approximately 10 million inhabitants), a network of research institutions started to collaborate in 2020 to develop a common analytical protocol based on an inter-calibration exercise between the network laboratories [14]. Although the results of this preliminary interlaboratory exercise had reassured the comparability of the results between the participating laboratories, the subsequent routine surveillance in the National Framework of the SARI WES project widely conducted in Italy [15] revealed differences between the results obtained by the different laboratories, which required further investigation. A new inter-calibration exercise was conducted among participating laboratories that aimed to establish a foundation for harmonized methodologies and to improve data comparability across laboratories.

## 2. Materials and Methods

### 2.1. Generation of Wastewater Samples Stock

**Figure 1:**
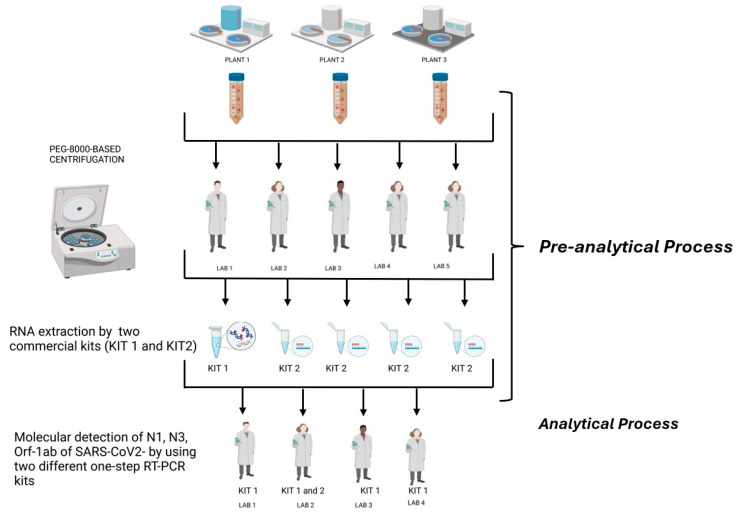
Workflow followed by the laboratories during the interlaboratory ring test (created in BioRender. https://BioRender.com/b09r836, accessed 14 February 2025).

Three composite 24 h raw, untreated urban wastewater samples were collected at the inlet of three different wastewater treatment plants (WWTPs) in the Lombardy region. The WWTPs are in a high-density urban setting in Milan, serving a population of more than 500,000 people each for the first two and about 300,000 people for the third, receiving mainly municipal waste (see Table 1). Sampling was performed in volume- or time-proportional mode, depending on the automatic sampler available. After the collection, samples were immediately processed for their viral concentration or were stored at −80 °C until analysis. The samples were collected in May 2022. These sewage samples were then split into 4 identical aliquots to be concentrated (pre-analytical phase) and tested (analytical phase) in parallel for the presence of SARS-CoV-2 RNA by carrying out real-time RT-PCR assays targeting the ORF-1ab and the N1 and N3 gene fragments in triplicate from four different laboratories (2 public health/virology university laboratories, 1 IZLER lab, and 1 laboratory at a WWTP). Regarding the pre-analytical phase, a fifth laboratory with extensive experience in the analytical determination of environmental samples was also included in the network. This laboratory participated in the interlaboratory comparison, specifically focusing on pre-analytical processes. The workflow followed by the laboratories during the interlaboratory ring test are illustrated in Figure 1.

### 2.2. Pre-Analytical Process: Concentration and Acid Nucleic Extraction

In compliance with the technical specifications outlined in EU Recommendation 2021/472, all participating laboratories in the nationwide SARI network implemented the same reference concentration protocol (ISS. SARI Rev.3 protocol, a protocol set up and used in a preliminary study conducted in Lombardy, [16] cfr. Castiglioni et al., 2022). Wastewater samples were subjected to the thermal inactivation of SARS-CoV-2 at 56 °C for 30 min. After cooling, 45 mL of each sample was concentrated using polyethylene glycol (PEG), and the samples were centrifuged at 4500× *g* for 30 min; subsequently, 40 mL of each sample was mixed with 8% (*w*/*v*) PEG 8000 and sodium chloride (0.3 M) (Sigma-Aldrich, St. Louis, MO, USA) and centrifuged at 12,000× *g* for 2 h. A process control virus (murine norovirus or mengovirus, provided by Istituto Superiore di Sanità (ISS, Rome, Italy) was added to each sample before concentration to monitor viral recovery from the samples, with the exception of one laboratory which was using a norovirus as an indicator of process control.

RNA was extracted by means of two commercial kits according to the manufacturer’s instructions and by combining two different protocols as follows: (1) QIAamp MinElute Virus Spin Kit (QIAGEN, Hilden, Germany) with an input of 400 μL of sample and an elution volume of 80 μL, as previously described. (B) NucliSens EasyMag (bioMerieux, Marcy-l’Étoile, France), with an input of 700 μL of sample and an elution volume of 100 μL.

### 2.3. Analytical Process: Real-Time One-Step RT-PCR Assays for SARS-CoV-2 Detection

The primer/probe sets used in this study targeted two different regions of the nucleocapsid (N) gene, namely N1 and N3, as listed by the CDC (USA) (2020), and ORF-1b-nsp14, according to the methods described by La Rosa and colleagues [17]. Two different one-step RT-PCR assays for SARS-CoV-2 were performed using the (1) AgPath-ID One-Step RT-PCR™ kit (Thermofisher Scientific, Waltham, MA, USA) and (2) QScript XLT 1-Step RT-PCR ToughMix^®^ (QuantaBio, Beverly, MA, USA). Primers and probes were obtained from Eurofins Genomics (Eurofins Genomics Germany GmbH, Ebersberg, Germany). To determine any potential contamination and/or inhibition, specific positive (EURM-019) and negative (DNAse/RNAse-free distilled water) controls were included in each real-time RT-PCR run. A sample was considered positive for SARS-CoV-2 when N1 or N3 or both viral targets showed a cycle threshold (Cq) ≤ 39. Real-time RT-PCR runs were performed by using the QuantStudio 5 Real-time RT-PCR system (Thermofisher Scientific, Waltham, MA, USA) and the CFX96 BIo-Rad Detection System (Bio-Rad, Milan, Italy). All samples were tested in triplicate and in three different runs.

In order to evaluate the analytical processes, to explore the SARS-CoV-2 RT-PCR assays’ performance, and to calibrate the RT-PCR methods, we constructed three standard curves as follows: (1) ORF-1ab targeted region was synthetized and purified by BioFab Research (Roma, Italy) and quantified by fluorometric measure (Qubit, Thermo Scientific) with a concentration of approximately 5 × 10^4^ copies/µL; (2) and (3) for N1 and N3 targeted gene fragments, the standard curves were constructed using the SARS-CoV-2 Research Grade Test Reference Material (RGTM 10169) from the National Institute of Standards and Technology (NIST), consisting of a synthetic RNA fragment from the SARS-CoV-2 genome with a concentration of approximately 5 × 10^6^ copies/µL [18].

Amplifications were considered acceptable if inhibition was ≤50% and if standard curves displayed a slope between −3.1 and −3.6 and an R^2^ ≥ 0.98 [19].

To minimize contamination risk, RNA extraction, molecular assays set up, and real-time RT-PCR runs were performed in separate rooms, according to the good laboratory practice for molecular assays.

### 2.4. Statistical Analyses

The statistical analysis was performed with IBM SPSS (ver. 28) using the log10-transform of the SARS-CoV-2 concentration (g.c./μL) as the response variable. The analysis was conducted separately for the ORF-1ab, N1, and N3 gene fragments. A family of Generalized Linear Models have been used to compare the results of the laboratories with different designs. A type III sum of squares approach was consistently used for the two-way ANOVA design within the GLM, as some cases involved unbalanced designs. The Bonferroni post hoc test was instead used to perform multiple pairwise comparisons among the laboratories.

#### 2.4.1. Pre-Analytical vs. Analytical Phase Effect

The GLM used to test the effect of the pre-analytical and analytical phases had the following design:

(1) $$y=\mu +\beta_{1}x_{Cq}+\beta_{2}A+\beta_{3}B+\beta_{4}AB+\varepsilon$$

where

y: response variable, log-transformed of the SARS-CoV-2 concentration measurement (g.c./L);

µ: true overall mean;

x_Cq_: covariate to account for the C_q_ (i.e., fractional PCR cycle used for quantification; cfr. [20]);

A: incremental effect of the laboratory responsible for the analytical phase (factor A);

B: incremental effect of the laboratory responsible for the pre-analytical phase (factor B);

AB: pre-analytical*analytical interaction term;

β_1_ is the coefficient for the covariate x_Cq_;

β_2_, β_3_, β_4_, are the coefficients associated with the categorical factors A and B and their interaction term, respectively;

ε: error term.

#### 2.4.2. Wastewater Treatment Plant Effect

A subsequent analysis incorporated the effect of the wastewater treatment plant using a GLM, which included three factors (wastewater treatment plant, pre-analytical, and analytical phases) considering only the WWTP*pre-analytical and WWTP*-analytical interaction terms.

(2) $$y=\mu +\beta_{1}x_{Cq}+\beta_{2}A+\beta_{3}B+\beta_{4}C+\beta_{5}AC+\beta_{6}BC+\varepsilon$$

where

y: response variable, log-transformed of the ith SARS-CoV-2 concentration measurement (g.c./L);

µ: true overall mean;

x_Cq_: covariate to account for the C_q_ (i.e., fractional PCR cycle used for quantification; cfr. [20]);

A: incremental effect of the laboratory responsible for the analytical phase (factor A);

B: incremental effect of the laboratory responsible for the pre-analytical phase (factor B);

C: incremental effect of the wastewater treatment plant (factor C); β_1_ is the coefficient for the covariate x_Cq_;

β_2_, β_3_, β_4_ are the coefficients associated with the categorical factors A, B, and C, respectively; β_5_, β_6_ are the coefficients associated with the interaction terms between categorical factors AC and BC;

ε: error term.

#### 2.4.3. RT-PCR Systems Effect

A third GLM analysis was used to compare the detections of SARS-CoV-2 by the the two different RT-PCR kits. The GLM included the Cq covariate, two factors (gene fragment, RT-PCR systems), and a gene fragment*RT-PCR system term.

(3) $$y=\mu +\beta_{1}x_{Cq}+\beta_{2}A+\beta_{3}B+\beta_{4}AB+\varepsilon \;$$

where

y: response variable, log-transformed of theSARS-CoV-2 concentration measurement (g.c./L);

µ: true overall mean; 

x_Cq_: covariate to account for the Cq (i.e., fractional PCR cycle used for quantification; cfr. [20]);

A: incremental effect of the gene fragment (factor A);

B: incremental effect of the RT-PCR systems (factor B);

AB: gene fragment*RT-PCR systems interaction term;

β_1_ is the coefficient for the covariate xCq;

β_2_, β_3_ are the coefficients associated with the categorical factors A and B, respectively;

β_4_ is the coefficient associated with the interaction terms between categorical factors AB;

ε: error term.

#### 2.4.4. Standard Curves Comparison

A fourth GLM analysis was used to compare the standard curves used by laboratories in the analytical phase. The GLM had the log-transformed Cq as the response variable, consisting of a covariate (log-transformed dilution factor), two factors (gene fragment, laboratory), and a gene fragment*analytical term.

(4) $$y=\mu +\beta_{1}x_{dil}+\beta_{2}A+\beta_{3}x_{dil}A+\varepsilon$$

where

y: response variable, log-transformed Cq (i.e., fractional PCR cycle used for quantification; cfr. [20]);

µ: true overall mean; 

x_dil_: covariate to account for the log-transformed dilution;

A: incremental effect of the laboratory responsible for the analytical phase (factor A);

x_dil_ A: dilution*analytical phase interaction term; β_1_ is the coefficient for the covariate x_dil_;

β_2_ is the coefficient associated with the categorical factor A;

β_3_ is the coefficient associated with the interaction term (x_dil_A) between covariate x_dil_ and factor A;

ε: error term.

#### 2.4.5. Frozen Samples Integrity

Viral gene copy counts from fresh and frozen samples (i.e., 24 samples were collected, with some aliquots analyzed fresh and others stored at −80 °C and analyzed after 12 months) were compared using Student’s paired *t*-test.

## 3. Results

### 3.1. Pre-Analytical vs. Analytical Phase

**Figure 2:**
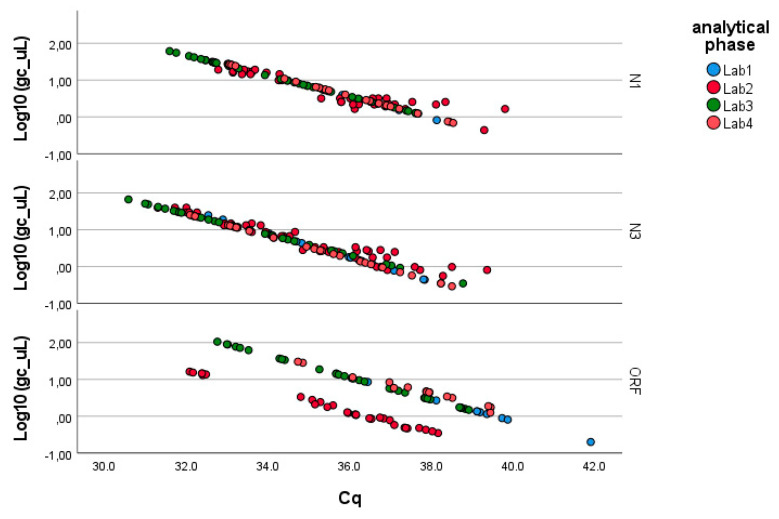
Linearized relationships of the log-transformed concentrations (g.c./μL) across the laboratories responsible for the analytical phase. Full regression statistics for the linear relationship depicted in the chart are provided in Table S1.

**Figure 3:**
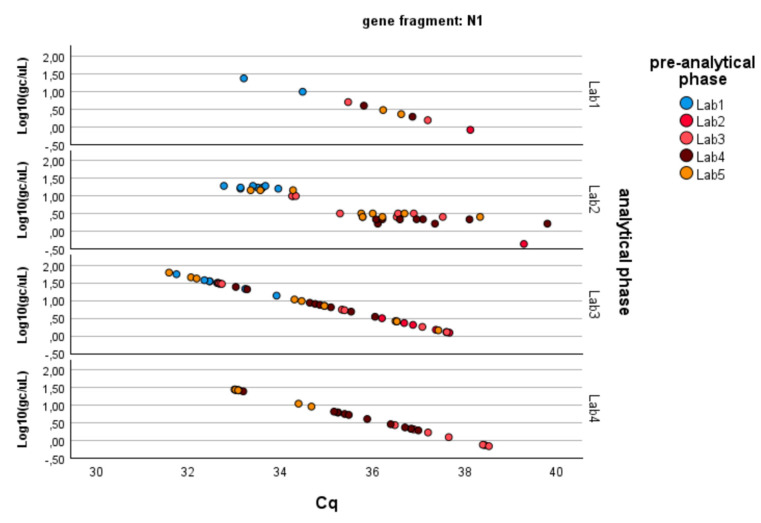
Log-transformed detections of N1 gene fragment copies/µL concentration: differences among laboratories concerning the analytical and pre-analytical phases. Full regression statistics for the linear relationship depicted in the chart are provided in Table S2.

As described in the Methods, the analysis was conducted separately for the three genes of SARS-CoV-2 (N1, N3, ORF1-ab; see Figure 2). Table 2 summarizes the results obtained through the application of Equation (1), which can be summarized as follows:

− N1 gene fragment: No factor is significant (*p*-value > 0.05). The laboratories demonstrate consistency in determining gene copies per liter, showing no variability due to the pre-analytical concentration/extraction processes or the analytical phase.
− N3 gene fragment: Significant differences are observed between laboratories in the analytical determination of gene copies per liter (*p*-value < 0.01), while no difference is present in the pre-analytical concentration and extraction processes (*p*-value > 0.20).
− ORF1-ab gene fragment: Significant differences are also observed here between laboratories in the analytical determination of gene copies per liter (*p*-value < 0.01), while no difference is present in the pre-analytical concentration and extraction processes (*p*-value > 0.40). Moreover, the pre-analytical*analytical interaction term is also significant (*p*-value < 0.01, see Figure 3). Pairwise multiple comparison tests of the analytical phase identified two laboratories (e.g., Lab2 and Lab4) as significantly different from the others (*p*-value < 0.05, see Table 3), particularly concerning the gene fragment ORF and, to a lesser extent, the gene fragment N3.

**Figure 4:**
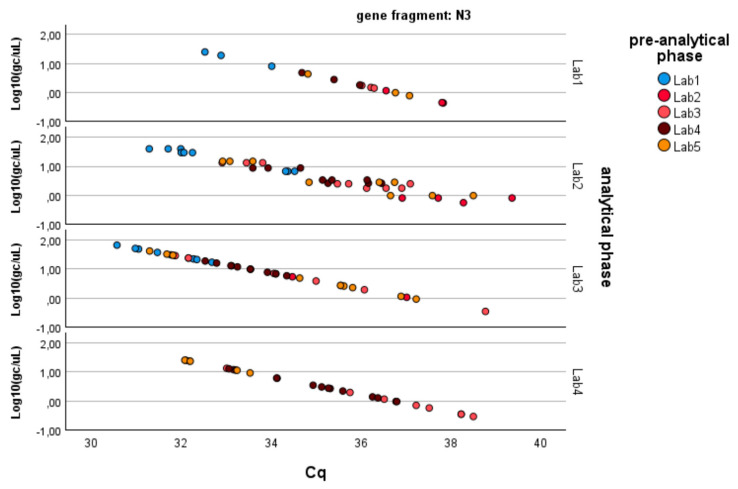
Log-transformed detections of N3 gene fragment copies/µL concentration: differences among laboratories concerning the analytical and pre-analytical phases. Full regression statistics for the linear relationship depicted in the chart are provided in Table S3.

**Figure 5:**
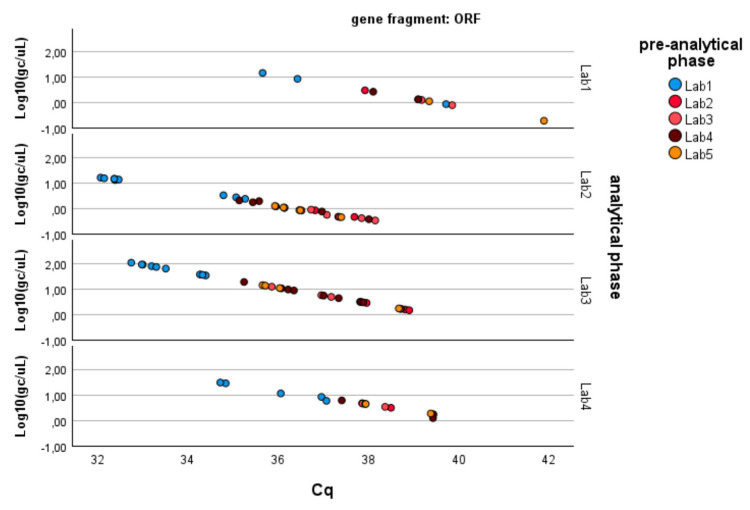
Log-transformed detections of ORF1ab gene fragment copies/µL concentration: differences among laboratories concerning the analytical and pre-analytical phases. Full regression statistics for the linear relationship depicted in the chart are provided in Table S4.

Since the pre-analytical*analytical interaction term was also found to be significant for the gene fragment ORF-1ab, a pairwise multiple comparison analysis was performed on this term, showing that Lab3 was the one showing significant differences (*p*-value < 0.05) among the pre-analytical sample extractions performed by different laboratories (Figure 3, Figure 4 and Figure 5).

### 3.2. Wastewater Treatment Plant Effect

After evaluating the pre-analytical and analytical phases, the effect of the WWTP was tested through the application of Equation (2). The wastewater treatment plant effect is significant (*p* < 0.001) only for the ORF-ab gene fragment, while it is not significant for the N1 or N3 genes (see Table 4).

**Figure 6:**
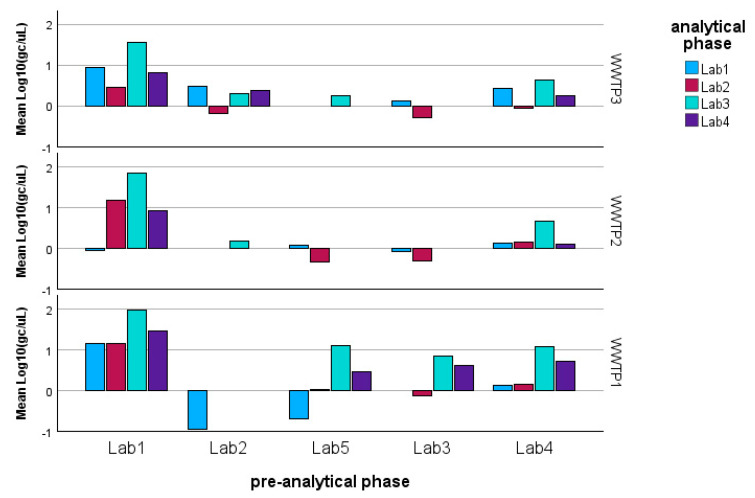
Model estimated marginal means of the log-transformed detections of ORF gene fragments: it can be observed that Lab2 shows the most significant variability with respect to the other laboratories and with respect to the WWTP.

It is also important to highlight the significant interaction term between the WWTP and the analytical phase for the ORFab gene. This interaction suggests that specific laboratories (namely Lab2 and Lab4) showed differing determination patterns for the smallest and largest WWTP (Figure 6). A similar effect was observed in the pre-analytical phase, where Lab2 likely encountered issues with the pre-analytical processing of the sample deriving from the largest WWTP.

### 3.3. RT-PCR Enzyme-Mix Effect

**Figure 7:**
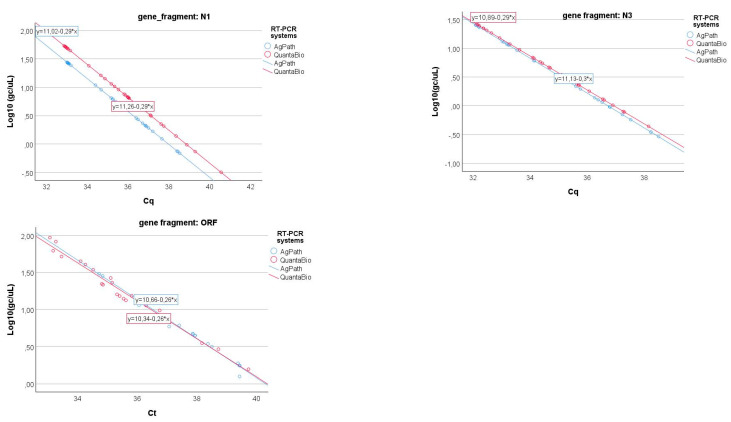
Linear relationships of the log-transformed gene copy values and Cq for the three gene fragments (N1, N3, and ORFab) of the two different RT-PCR systems (e.g., AgPath and QuantaBio).

One of the laboratories conducted the analysis using two different one-step RT-PCR assays for SARS-CoV-2: (1) the AgPath-ID One-Step RT-PCR™ Kit (Thermo Fisher Scientific, Waltham, MA, USA) and (2) the QScript XLT 1-Step RT-PCR ToughMix^®^ (QuantaBio, Beverly, MA, USA). The results obtained from the same samples were compared through a specific GLM (cfr. Equation (3)), and the results are shown in Figure 7. As can be seen in Table 5, only the N3 gene fragment shows a significant difference in the slopes (i.e., the coefficient of gene fragment*RT-PCR systems interaction term) of the relationship between the log-transformed gene copy values and Cq, while this term is not significant for the two other gene fragments.

### 3.4. Harmonization of Standard Curves Across Laboratories

**Figure 8:**
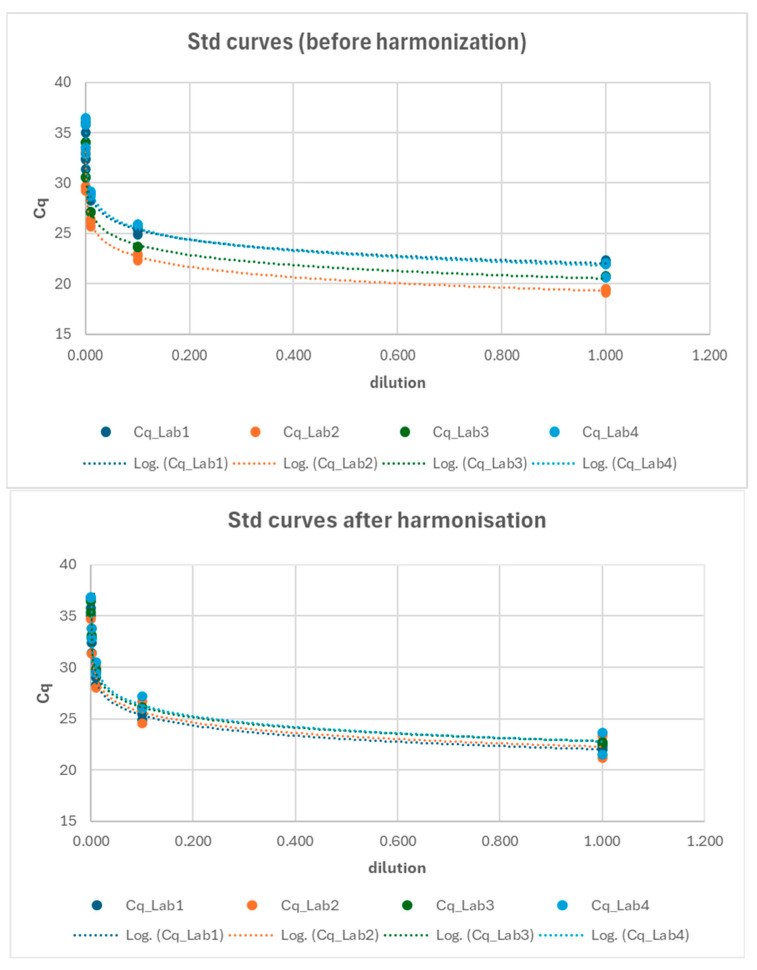
Interlaboratory comparison of standard curves before (**upper chart**) and after (**lower chart**) the harmonization process.

After identifying the primary issue in the analytical phase for the ORF gene, an effort was made to reverify the standardization of protocols, and the reference standards for the ORF gene were redistributed to ensure that all laboratories had the opportunity to reconstruct their standard curves accordingly. The standard curves from the three laboratories were compared at two key points: (1) before the harmonization and (2) after the harmonization process (Figure 8). A Generalized Linear Model (GLM) approach (cfr. Equation (4)) was employed to compare the relationship between log-transformed Cq values and log-transformed dilutions across the laboratories. The results presented in Table 6 indicate that, following harmonization, all the standard curves were comparable across the laboratories (e.g., Lab*log_dilution interaction not significant; see also Figure S1).

### 3.5. SARS-CoV-2 Detection in Frozen Samples

The main objective of this analysis was to evaluate whether freezing samples at −80 °C for 12 months preserved the integrity of the gene copy determination. To achieve this, gene copy counts of SARS-CoV-2 from fresh samples were compared with those from the corresponding frozen samples using Student’s paired *t*-test. As shown in Table 7, the paired *t*-test results clearly indicate a significant difference in gene copy determination after freezing and storing the samples. The analysis of the gene copy ratios between fresh and frozen samples (Table 8) indicates that freezing may lead to a loss of up to 6 logs in SARS-CoV-2 enumeration, primarily affecting the N1 and N3 gene fragments; however, in most cases, the median loss is of the order of just a few units.

## 4. Discussion

Urban wastewater is an immediate by-product of human activities in urban environments, and its composition mirrors the presence and concentrations of microbiological, chemical, and physical pollutants associated with the population. In recent years, an increasing body of evidence has highlighted the potential of wastewater surveillance as a valuable tool for detecting and monitoring circulating pathogens within communities [21,22,23].

During the COVID-19 pandemic, the use of WES for SARS-CoV-2 and its variants has emerged as a powerful complementary tool to clinical epidemiology, providing valuable and timely insights into the virus’s presence and circulation within a certain community [24]. Moreover, several laboratories have shown that the detection of new SARS-CoV-2 variants in wastewater samples precedes its identification at the clinical level [23,24,25,26], emphasizing WES as a proactive and early warning system [27]. However, analyzing the results using high-throughput sequencing approaches requires sophisticated data analysis tools and specialized expertise and resources. In addition, sample quality and variability in wastewater composition can affect detection sensitivity and specificity, presenting challenges in tracking shifts in SARS-CoV-2 sequences across different regions and over time [28]. A considerable number of assays have been published in the scientific literature for the detection of SARS-CoV-2 in wastewater. Furthermore, a number of papers have evaluated the efficacy of different procedures applied in the analytical workflow [7,8,29,30,31,32,33]. Recent review studies have highlighted the importance of continuously monitoring the performance of existing assays, as well as the necessity of developing new, more effective assays [10,34]. Although most national laboratories have implemented internal and sometimes external quality control measures to ensure the efficient execution of their workflows, a uniform and standardized approach remains elusive [10]. Moreover, to date, no International External Quality Assessment (EQA) nor proficiency testing (PT) program within the wastewater molecular diagnostics area have been implemented. However, monitoring SARS-CoV-2 in wastewater systems poses significant challenges [11,12], and the lack of standardized methods for assessing viral RNA concentration, extraction, and quantification in the WES of SARS-CoV-2 may complicate the comparison of data among laboratories [11,12,13], limiting the effectiveness and reliability of WES monitoring [13]. Recent literature reviews [10,35,36] have reported heterogeneity and a lack of best practices concerning analytical procedures in SARS-CoV-2 WES, reinforcing the need for higher standards and quality controls to improve the accuracy of results and promote harmonization and data comparability.

Our study demonstrates that a diverse set of methods, targeting different gene fragments, enables quantifying the SARS-CoV-2 genetic signal in raw wastewater samples collected from high-density urban areas with high reproducibility. In fact, 78% of the data from the four different laboratories fell within a range of approximately ±1 log10 gc/uL, with a few data exceeding the threshold of 1 log10 gc/uL concerning the ORF-ab gene fragment and the largest WWTP (1,250,000 PE). Additionally, the interlaboratory analysis workflow and the design of strong statistical methods facilitated the identification of the primary sources of variability between the analytical and pre-analytical phases involved in the quantification of SARS-CoV-2. These sources of variability were found to be significantly higher for the ORF-ab gene fragments compared to the N1 and N3 gene fragments, with the analytical phase identified as being more influential than the pre-analytical phase.

Moreover, the analysis of the same gene fragment was influenced by the variability of the wastewater source (e.g., the WWTP from which the sample was collected). Some laboratories exhibited different determination patterns between the smallest and largest WWTPs. A similar effect was observed during the pre-analytical phase, where Lab2 likely encountered challenges in processing samples originating from the largest WWTP. The potential variability arising from the use of different RT-PCR commercial enzymes kits was also tested, revealing a very limited effect on SARS-CoV-2 detection. The reasons for these results are not clear; however, it could be hypothesized that in the wastewater of larger plants, there may be more interfering factors than in small ones.

These results highlighted that the primary source of variability in the detections was associated with the analytical phase, most likely linked to the standard curves used by the different laboratories. Therefore, these curves were harmonized across the laboratories, providing a consistent standard for all. Following this harmonization, the associated variability factor was eliminated. Finally, the integrity of the frozen samples was assessed by comparing the SARS-CoV-2 enumeration of the corresponding fresh and frozen samples. The results showed that freezing could lead to a loss of up to 6 logs in SARS-CoV-2 enumeration, primarily affecting the N1 and N3 gene fragments. However, in most cases, the median loss was limited to just a few gene copy units per microL.

Although the accuracy of the different determinations—specifically, their ability to correctly quantify the true number of SARS-CoV-2 genome copies—could not be assessed due to the unknown concentrations in the raw wastewater samples; these results are highly encouraging and emphasize the significance of interlaboratory comparison studies.

A lack of standardized protocols and harmonized quality assurance and quality control (QA/QC) procedures underscores the necessity for certified reference materials in this domain since while laboratories currently employ control materials, their use tends to be fragmented, heterogeneous, and limited to specific steps within the analytical workflow. As Paracchini and colleagues [10] demonstrated in their EU survey, there is a lack of consensus on the optimal WES normalization parameters (e.g., for controlling the RNA extraction step and/or fecal content), which are essential for establishing more robust correlations between SARS-CoV-2 WES data and other traditional clinical indicators.

The use of surrogate viruses, which is a common practice, also has its limitations, as these viruses do not always accurately replicate the physical and chemical characteristics of SARS-CoV-2 [37].

In general, there is a critical gap and an urgent need for improvement, especially in the development of reference materials certified for copy number concentration and sequence identity, ideally closely related to the target virus. The introduction of such materials, used as spikes at the initial steps of the process, could significantly enhance the accuracy of the pathogen recovery rate calculations. Additionally, the use of fecal controls would ensure accurate data interpretation and comparison, accounting for variations in matrix composition and providing more reliable insights into viral presence and trends. The progress toward the development of such reference standards and materials is hindered by the fact that proficiency testing or ring tests remain relatively rare among laboratories implementing WES.

All this underscores the necessity of organizing proficiency tests (PTs) to assess laboratory performance, identify potential gaps, uncover best practices, and promote continuous improvement in the quality of laboratory capabilities. Harmonized results among laboratories analyzing wastewater samples are in fact crucial to align temporal trends, ensuring quantitative data expressed in a uniform unit. This standardization is pivotal for establishing a comprehensive overview, especially in the face of emerging viruses, enabling effective monitoring and response across diverse laboratories and countries.

This is even more important as environmental surveillance continues to gain significance, not only through the wastewater-based methods explored in this study but also through emerging innovative approaches such as Human Biomonitoring [38] and nanoscale microscopy [39]. These advanced techniques enable the ultra-sensitive detection of target signals in water resources, further enhancing early warning capabilities and supporting more effective public health interventions. The lessons learned from this interlaboratory comparison study, along with the experimental set up and statistical analysis approach, may contribute to the development and standardization of emerging innovative techniques, further enhancing the sensitivity and reliability of environmental surveillance in water resources.

## 5. Conclusions

Multiple laboratories are required to conduct WES across extensive areas, but even when they share the same analytical procedure, they can still be a significant source of variability.

Interlaboratory comparison studies may greatly help to identify discrepancies and errors in analytical results, which may arise from various factors, or identify the most significant sources of variation.

The diverse approaches still employed across various steps in WES workflows highlight the ongoing challenges of harmonization, underscoring the need for standardized methodologies and reference materials.

Amid such a diversity of approaches, interlaboratory comparisons play a vital role in evaluating the reliability and consistency of analytical methods across laboratories. To maximize their effectiveness, it is essential that these trials are conducted regularly and adhere to a standardized framework. This framework should comprehensively address all stages, from the analytical protocol to statistical analysis, ensuring robust, reproducible results while identifying and addressing potential sources of variability.

Harmonized results among laboratories are in fact essential for ensuring consistency in temporal trend analysis, facilitating the expression of quantitative data in standardized units, and enabling the integration of findings into a comprehensive overview. This uniformity is particularly critical when addressing emerging viruses as it allows for reliable comparisons across different laboratories and countries. By aligning methodologies and standardizing data reporting, laboratories can enhance the accuracy of surveillance efforts, improve early detection capabilities, and support a more effective and coordinated public health response. Collaborative initiatives, such as the EU-WISH Joint Action under the EU4Health program, play a crucial role in strengthening the European Union’s capacity to prevent, prepare for, and respond rapidly to serious cross-border health threats. These efforts are particularly valuable in enhancing national capabilities for wastewater-based public health surveillance by fostering knowledge exchange, promoting the adoption of best practices, and ensuring that methodologies are grounded in robust scientific evidence. By facilitating cooperation among countries, such initiatives contribute to more effective and coordinated public health responses, ultimately improving preparedness and resilience against emerging health risks.

## Figures and Tables

**Table 1:** WWTP flows and water quality (standard deviations, SDs, between brackets).

Parameter	Plant1	Plant2	Plant3
People Equivalent	1,250,000	600,000	296,000
Annual Average flow (m^3^/d)	432,000	179,000	75,000
TSSs (mg/L)	225.0 (±58.1)	179.8 (±102.8)	209.0 (±90.6)
BOD (mg/L)	210.2 (±54.6)	246.0 (±103.6)	
COD (mg/L)	384.9 (±100.0)	374.2 (±164.4)	428.1 (±142.8)
N-NH4 (mg/L)	21.0 (±4.6)	28.8 (±6.1)	43.9 (±9.5)
N-tot (mg/L)	30.9 (±5.7)	34.0 (±6.2)	44.0 (±7.8)

**Table 2:** Generalized Linear Model tests of between-subject effects: effect size of the pre-analytical and analytical phases and their interaction.

Dependent Variable: Log10-Transform of the SARS-CoV-2 Concentration (g.c./μL)						
Gene	Source	Sum of Squares	df	Mean Square	F	*p*-Value
N1 _a_	Intercept	13.164	1	13.164	958.175	<0.001
	Cq	11.393	1	11.393	829.310	<0.001
	Analytical phase	0.044	3	0.015	1.078	0.362
	Pre-analytical phase	0.068	4	0.017	1.235	0.301
	Pre-analytical * Analytical	0.051	11	0.005	0.337	0.975
	Error	1.333	97	0.014		
N3 _b_	Intercept	18.798	1	18.798	2040.562	<0.001
	Cq	16.565	1	16.565	1798.146	<0.001
	Analytical phase	0.411	3	0.137	14.859	<0.001
	Pre-analytical phase	0.051	4	0.013	1.391	0.242
	Pre-analytical * Analytical	0.122	10	0.012	1.325	0.227
	Error	0.940	102	0.009		
ORF1ab _c_	Intercept	6.397	1	6.397	5360.707	<0.001
	Cq	5.890	1	5.890	4935.942	<0.001
	Analytical phase	11.139	3	3.713	3111.654	<0.001
	Pre-analytical phase	0.005	4	0.001	0.998	0.415
	Pre-analytical * Analytical	0.052	12	0.004	3.650	<0.001
	Error	0.080	67	0.001		

_a_. R^2^ = 0.957 (adjusted R^2^ = 0.948). _b_. R^2^ = 0.978 (adjusted R^2^ = 0.974). _c_. R^2^ = 0.998 (adjusted R^2^ = 0.997).

**Table 3:** Pairwise multiple comparisons: differences between laboratories concerning the analytical phase. The mean difference is estimated based on the GLM marginal means, and the significance level is adjusted for multiple comparisons through Bonferroni’s correction.

Dependent Variable: Log10-Transform of the SARS-CoV-2 Concentration (g.c./μL)							
Gene Fragment	(I) Lab	(J) Lab	Mean Difference (I-J)	Std. Error	*p*-Value	95% Confidence Interval for Difference	
						Lower Bound	Upper Bound
N3	LAB1	LAB2	−0.149 *	0.030	<0.001	−0.229	−0.070
		LAB3	−0.061	0.032	0.348	−0.146	0.025
		LAB4	0.007a	0.032	1.000	−0.079	0.093
	LAB2	LAB1	0.149 *	0.030	<0.001	0.070	0.229
		LAB3	0.088 *	0.025	0.003	0.021	0.155
		LAB4	0.156 *	0.025	<0.001	0.088	0.224
	LAB3	LAB1	0.061	0.032	0.348	−0.025	0.146
		LAB2	−0.088 *	0.025	0.003	−0.155	−0.021
		LAB4	0.068a	0.026	0.055	−0.001	0.137
	LAB4	LAB1	−0.007	0.032	1.000	−0.093	0.079
		LAB2	−0.156 *	0.025	<0.001	−0.224	−0.088
		LAB3	−0.068	0.026	0.055	−0.137	0.001
ORF	LAB1	LAB2	0.894 *	0.017	<0.001	0.846	0.941
		LAB3	−0.023	0.016	0.935	−0.067	0.021
		LAB4	−0.170 *	0.015	<0.001	−0.211	−0.129
	LAB2	LAB1	−0.894 *	0.017	<0.001	−0.941	−0.846
		LAB3	−0.917 *	0.010	<0.001	−0.945	−0.889
		LAB4	−1.063 *	0.015	<0.001	−1.103	−1.024
	LAB3	LAB1	0.023	0.016	0.935	−0.021	0.067
		LAB2	0.917 *	0.010	<0.001	0.889	0.945
		LAB4	−0.146 *	0.013	<0.001	−0.183	−0.110
	LAB4	LAB1	0.170 *	0.015	<0.001	0.129	0.211
		LAB2	1.063 *	0.015	<0.001	1.024	1.103
		LAB3	0.146 *	0.013	<0.001	0.110	0.183

* difference significant at *p* < 0.05 level.

**Table 4:** Generalized Linear Model tests of between-subject effects: effect size of WWTP, pre-analytical and analytical phases, and their interactions.

Dependent Variable: Log10-Transform of the SARS-CoV-2 Concentration (g.c./μL)						
Gene	Source	Sum of Squares	df	Mean Square	F	*p*-Value
N1 _a_	Intercept	7.680	1	7.680	559.595	<0.001
	Cq	6.560	1	6.560	477.987	<0.001
	WWTP	0.037	2	0.019	1.354	0.263
	Analytical phase	0.043	3	0.014	1.054	0.372
	Pre-analytical phase	0.116	4	0.029	2.108	0.086
	WWTP*Analytical	0.029	5	0.006	0.424	0.831
	WWTP*Pre-analytical	0.038	8	0.005	0.345	0.946
	Error	1.276	93	0.014		
N3 _b_	Intercept	8.095	1	8.095	806.021	<0.001
	Cq	7.126	1	7.126	709.460	<0.001
	WWTP	0.008	2	0.004	0.412	0.663
	Analytical phase	0.381	3	0.127	12.630	<0.001
	Pre-analytical phase	0.075	4	0.019	1.859	0.124
	WWTP*Analytical	0.039	6	0.007	0.651	0.689
	WWTP*Pre-analytical	0.054	8	0.007	0.672	0.715
	Error	0.964	96	0.010		
ORFab _c_	Intercept	3.710	1	3.710	8012.754	<0.001
	Ct	3.497	1	3.497	7551.102	<0.001
	WWTP	0.025	2	0.012	26.608	<0.001
	Analytical phase	10.649	3	3.550	7665.489	<0.001
	Pre-analytical phase	0.001	4	0.000	0.626	0.646
	WWTP*Analytical	0.057	6	0.010	20.656	<0.001
	WWTP*Pre-analytical	0.002	7	0.000	0.476	0.848
	Error	0.030	64	0.000		

_a_. R^2^ = 0.959 (adjusted R^2^ = 0.948). _b_. R^2^ = 0.977 (adjusted R^2^ = 0.971). _c_. R^2^ = 0.999 (adjusted R^2^ = 0.999).

**Table 5:** GLM between-subject effects considering the effect of the different RT-PCR systems (e.g., Quantabio and AgPath).

Dependent Variable: Log10-Transform of the SARS-CoV-2 Concentration (g.c./μL)						
Gene Fragment	Source	Sum of Squares	df	Mean Square	F	*p*-Value
N1 _a_	Cq	19.609	1	19.609	16,362,402.024	<0.001
	RT-PCR system	0.003	1	0.003	2368.974	<0.001
	RT-PCR system*Cq	2.092 × 10^−7^	1	2.092 × 10^−7^	0.175	0.678
	Error	6.591 × 10^−5^	55	1.198 × 10^−6^		
N3 _b_	Ct	17.698	1	17.698	12,665,566.600	<0.001
	RT-PCR system	0.002	1	0.002	1645.421	<0.001
	RT-PCR system*Cq	0.003	1	0.003	2340.033	<0.001
	Error	7.406 × 10^−5^	53	1.397 × 10^−6^		
ORF _c_	Ct	6.054	1	6.054	1651.323	<0.001
	RT-PCR system	0.002	1	0.002	0.450	0.507
	RT-PCR system*Cq	0.002	1	0.002	0.416	0.523
	Error	0.125	34	0.004		

_a_. R^2^ = 1.000 (adjusted R^2^ = 1.000). _b_. R^2^ = 1.000 (adjusted R^2^ = 1.000). _c_. R^2^ = 0.987 (adjusted R^2^ = 0.986).

**Table 6:** Standard curve comparison after harmonization.

Dependent Variable: Log-Tranformed (Cq)						
Gene Fragment	Source	Sum of Squares	df	Mean Square	F	*p*-Value
N1 _a_	log_dilution	0.072	1	0.072	23.577	<0.001
	Lab	0.002	3	0.001	0.256	0.855
	Lab*log_dilution	0.000	3	6.721 × 10^−5^	0.022	0.995
	Error	0.033	11	0.003		
N3 _b_	log_dilution	0.073	1	0.073	23.793	<0.001
	Lab	0.002	3	0.001	0.231	0.873
	Lab*log_dilution	0.001	3	0.000	0.056	0.982
	Error	0.034	11	0.003		
ORF _c_	log_dilution	0.150	1	0.150	66.223	<0.001
	Lab	0.013	3	0.004	1.945	0.137
	Lab*log_dilution	0.001	3	0.000	0.110	0.954
	Error	0.095	42	0.002		

_a_. R^2^ = 0.697 (adjusted R^2^ = 0.504). _b_. R^2^ = 0.697 (adjusted R^2^ = 0.504). _c_. R^2^ = 0.688 (adjusted R^2^ = 0.636).

**Table 7:** Student’s paired *t*-test results of the comparison between fresh and frozen samples.

Gene		Paired Differences							
		Mean	Std. Dev	Std. Error	95% Confidence Interval of the Difference		t	df	*p*-Value
					Lower	Upper			
N1	T0–T1	30,106.08	38,406.66	7839.73	13,888.37	46,323.79	3.840	23	0.001
N3	T0–T1	13,108.94	29,665.32	6055.41	582.37	25,635.50	2.165	23	0.041
ORF	T0–T1	10,090.75	22,711.99	4636.07	500.32	19,681.19	2.177	23	0.040

**Table 8:** Quartiles of the gene copy ratios between fresh and frozen samples.

N1	N		24
	Percentiles	25	3.5
		50	13.7
		75	6,512,257.1
N3	N		24
	Percentiles	25	1.4
		50	4.6
		75	1,746,123.1
ORF	N		24
	Percentiles	25	1.0
		50	1.0
		75	12.6

## Data Availability

Data supporting the findings of this study are available from corresponding author upon reasonable request.

## Supplementary Materials

The following supporting information can be downloaded at: https://www.mdpi.com/article/10.3390/microorganisms13030526/s1, Figure S1. Weekly SARS-CoV-2 concentrations in wastewater following the harmonization of standard curves across laboratories. Surveillance conducted during the winter months (weeks 1–13) showed strong alignment among laboratories, particularly when different laboratories sampled the same WWTP on different days within the same week. Table S1. Full regression statistics for the linear relationships depicted in Figure 2. Table S2. Full regression statistics for the linear relationships for gene fragment N1, shown in Figure 3. Table S3. Full regression statistics for the linear relationships for gene fragment N3, shown in Figure 4. Table S4. Full regression statistics for the linear relationships for gene fragment ORF1ab shown in Figure 5.

## Abbreviations

The following abbreviations are used in this manuscript:

WES	Wastewater environmental surveillance
PE	People Equivalent
GLMs	Generalized Linear Models
WWTP	Wastewater treatment plant
QCMD	Quality Control for Molecular Diagnostics
TSSs	Total Suspended Solids
BOD	Biochemical Oxygen Demand
COD	Chemical Oxygen Demand
NIST	National Institute of Standards and Technology
