# Supplementary material for: Evaluating Interlaboratory Variability in Wastewater-Based COVID-19 Surveillance
Source: Microorganisms. 2025 Feb 27;13(3):526. doi: 10.3390/microorganisms13030526 (PMC11945948; doi:10.3390/microorganisms13030526)
Supplement: Supplementary file 1 [file microorganisms-13-00526-s001.zip › Figure S1.docx]

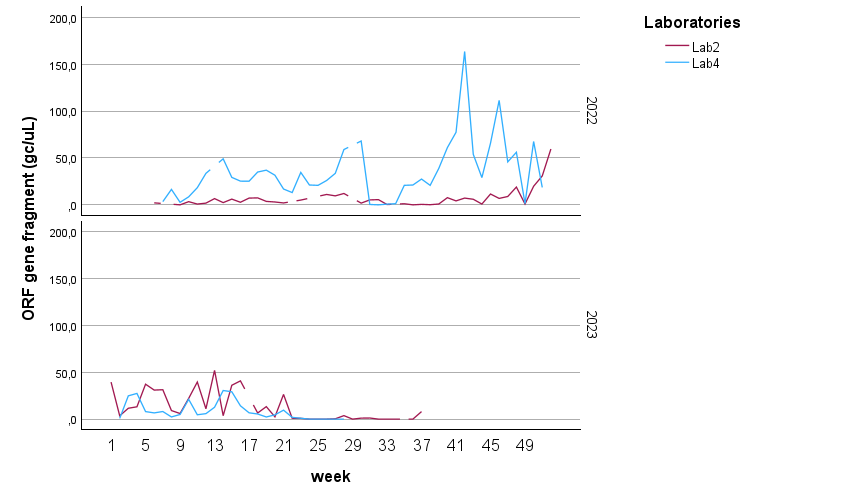


***Figure S1.*** Weekly SARS-CoV-2 concentrations in wastewater following the harmonization of standard curves across laboratories. Surveillance conducted during the winter months (weeks 1–13) showed strong alignment among laboratories, particularly when different laboratories sampled the same WWTP on different days within the same week.
