# Supplementary material for: Evaluating Interlaboratory Variability in Wastewater-Based COVID-19 Surveillance
Source: Microorganisms. 2025 Feb 27;13(3):526. doi: 10.3390/microorganisms13030526 (PMC11945948; doi:10.3390/microorganisms13030526)
Supplement: Supplementary file 1 [file microorganisms-13-00526-s001.zip › TableS1.docx]

***Table S1.*** Full regression statistics for the linear relationships depicted in Figure 2

|  |  |  | | **B** | **R-square** |
| --- | --- | --- | --- | --- | --- |
| N1 | Lab1 |  | (Constant) | 11.201 | 0.999 |
|  |  |  | Cq | -0.296 |  |
|  | Lab2 |  | (Constant) | 8.443 | 0.846 |
|  |  |  | Cq | -0.218 |  |
|  | Lab3 |  | (Constant) | 10.603 | 0.999 |
|  |  |  | Cq | -0.279 |  |
|  | Lab4 |  | (Constant) | 11.017 | 0.999 |
|  |  |  | Cq | -0.290 |  |
| N3 | Lab1 |  | (Constant) | 12.167 | 0.999 |
|  |  |  | Cq | -0.331 |  |
|  | Lab2 |  | (Constant) | 9.505 | 0.929 |
|  |  |  | Cq | -0.252 |  |
|  | Lab3 |  | (Constant) | 10.356 | 0.999 |
|  |  |  | Cq | -0.279 |  |
|  | Lab4 |  | (Constant) | 11.130 | 0.999 |
|  |  |  | Cq | -0.303 |  |
| ORF | Lab1 |  | (Constant) | 11.824 | 0.999 |
|  |  |  | Cq | -0.299 |  |
|  | Lab2 |  | (Constant) | 10.327 | 0.993 |
|  |  |  | Cq | -0.284 |  |
|  | Lab3 |  | (Constant) | 11.884 | 0.999 |
|  |  |  | Cq | -0.301 |  |
|  | Lab4 |  | (Constant) | 10.657 | 0.999 |
|  |  |  | Cq | -0.264 |  |
