# Supplementary material for: Evaluating Interlaboratory Variability in Wastewater-Based COVID-19 Surveillance
Source: Microorganisms. 2025 Feb 27;13(3):526. doi: 10.3390/microorganisms13030526 (PMC11945948; doi:10.3390/microorganisms13030526)
Supplement: Supplementary file 1 [file microorganisms-13-00526-s001.zip › TableS2.docx]

***Table S2.*** Full regression statistics for the linear relationships for gene fragment N1, shown in Figure 3

|  |  |  | | **B** | **R-square** |
| --- | --- | --- | --- | --- | --- |
| **Lab1** | **Lab2** |  | (Constant) | 11.201 | 0.999 |
|  |  |  | Cq | -0.296 |  |
|  | **Lab5** |  | (Constant) | 11.201 | 0.999 |
|  |  |  | Cq | -0.296 |  |
|  | **Lab3** |  | (Constant) | 11.201 | 0.999 |
|  |  |  | Cq | -0.296 |  |
|  | **Lab4** |  | (Constant) | 11.201 | 0.999 |
|  |  |  | Cq | -0.296 |  |
| **Lab2** | **Lab1** |  | (Constant) | 2.316 | 0.110 |
|  |  |  | Cq | -0.032 |  |
|  | **Lab5** |  | (Constant) | 70.646 | 0.764 |
|  |  |  | Cq | -0.196 |  |
|  | **Lab3** |  | (Constant) | 7.521 | 0.761 |
|  |  |  | Cq | -0.193 |  |
|  | **Lab4** |  | (Constant) | 1.036 | 0.148 |
|  |  |  | Cq | -0.020 |  |
| **Lab3** | **Lab1** |  | (Constant) | 10.603 | 0.999 |
|  |  |  | Cq | -0.279 |  |
|  | **Lab2** |  | (Constant) | 10.603 | 0.999 |
|  |  |  | Cq | -0.279 |  |
|  | **Lab5** |  | (Constant) | 10.603 | 0.999 |
|  |  |  | Cq | -0.279 |  |
|  | **Lab3** |  | (Constant) | 10.603 | 0.999 |
|  |  |  | Cq | -0.279 |  |
|  | **Lab4** |  | (Constant) | 10.603 | 0.999 |
|  |  |  | Cq | -0.279 |  |
| **Lab4** | **Lab1** |  | (Constant) | 10.390 | 0.999 |
|  |  |  | Cq | -0.271 |  |
|  | **Lab5** |  | (Constant) | 10.988 | 0.999 |
|  |  |  | Cq | -0.289 |  |
|  | **Lab3** |  | (Constant) | 11.027 | 0.999 |
|  |  |  | Cq | -0.290 |  |
|  | **Lab4** |  | (Constant) | 11.028 | 0.999 |
|  |  |  | Cq | -0.290 |  |
