# Supplementary material for: Evaluating Interlaboratory Variability in Wastewater-Based COVID-19 Surveillance
Source: Microorganisms. 2025 Feb 27;13(3):526. doi: 10.3390/microorganisms13030526 (PMC11945948; doi:10.3390/microorganisms13030526)
Supplement: Supplementary file 1 [file microorganisms-13-00526-s001.zip › TableS3.docx]

***Table S3.*** Full regression statistics for the linear relationships for gene fragment N3, shown in Figure 4

|  |  |  | | **B** | **R-square** |
| --- | --- | --- | --- | --- | --- |
| **Lab1** | **Lab1** |  | (Constant) | 12.167 | 0.999 |
|  |  |  | Cq | -0.331 |  |
|  | **Lab2** |  | (Constant) | 12.167 | 0.999 |
|  |  |  | Cq | -0.331 |  |
|  | **Lab5** |  | (Constant) | 12.167 | 0.999 |
|  |  |  | Cq | -0.331 |  |
|  | **Lab3** |  | (Constant) | 12.167 | 0.999 |
|  |  |  | Cq | -0.331 |  |
|  | **Lab4** |  | (Constant) | 12.167 | 0.999 |
|  |  |  | Cq | -0.331 |  |
| **Lab2** | **Lab1** |  | (Constant) | 10.294 | 0.977 |
|  |  |  | Cq | -0.275 |  |
|  | **Lab2** |  | (Constant) | 0.275 | 0.019 |
|  |  |  | Cq | -0.011 |  |
|  | **Lab5** |  | (Constant) | 8.956 | 0.880 |
|  |  |  | Cq | -0.236 |  |
|  | **Lab3** |  | (Constant) | 9.207 | 0.885 |
|  |  |  | Cq | -0.244 |  |
|  | **Lab4** |  | (Constant) | 8.018 | 0.759 |
|  |  |  | Cq | -0.210 |  |
| **Lab3** | **Lab1** |  | (Constant) | 10.363 | 0.999 |
|  |  |  | Cq | -0.279 |  |
|  | **Lab2** |  | (Constant) | 10.346 | 0.999 |
|  |  |  | Cq | -0.279 |  |
|  | **Lab5** |  | (Constant) | 10.363 | 0.999 |
|  |  |  | Cq | -0.279 |  |
|  | **Lab3** |  | (Constant) | 10.344 | 0.999 |
|  |  |  | Cq | -0.279 |  |
|  | **Lab4** |  | (Constant) | 10.363 | 0.999 |
|  |  |  | Cq | -0.279 |  |
| **Lab4** | **Lab5** |  | (Constant) | 11.153 | 0.999 |
|  |  |  | Cq | -0.304 |  |
|  | **Lab3** |  | (Constant) | 11.128 | 0.999 |
|  |  |  | Cq | -0.303 |  |
|  | **Lab4** |  | (Constant) | 11.127 | 0.999 |
|  |  |  | Cq | -0.303 |  |
