# Supplementary material for: Evaluating Interlaboratory Variability in Wastewater-Based COVID-19 Surveillance
Source: Microorganisms. 2025 Feb 27;13(3):526. doi: 10.3390/microorganisms13030526 (PMC11945948; doi:10.3390/microorganisms13030526)
Supplement: Supplementary file 1 [file microorganisms-13-00526-s001.zip › TableS4.docx]

***Table S4.*** Full regression statistics for the linear relationships for gene fragment ORF1ab. shown in Figure 5

|  |  |  | | **B** | **R-square** |
| --- | --- | --- | --- | --- | --- |
| **Lab1** | **Lab1** |  | (Constant) | 11.824 | 0.999 |
|  |  |  | Cq | -0.299 |  |
|  | **Lab5** |  | (Constant) | 11.824 | 0.999 |
|  |  |  | Cq | -0.299 |  |
|  | **Lab3** |  | (Constant) | 11.824 | 0.999 |
|  |  |  | Cq | -0.299 |  |
|  | **Lab4** |  | (Constant) | 11.824 | 0.999 |
|  |  |  | Cq | -0.299 |  |
| **Lab2** | **Lab1** |  | (Constant) | 9.551 | 0.999 |
|  |  |  | Cq | -0.260 |  |
|  | **Lab2** |  | (Constant) | 10.873 | 0.999 |
|  |  |  | Cq | -0.297 |  |
|  | **Lab5** |  | (Constant) | 10.726 | 0.999 |
|  |  |  | Cq | -0.296 |  |
|  | **Lab3** |  | (Constant) | 9.169 | 0.964 |
|  |  |  | Cq | -0.253 |  |
|  | **Lab4** |  | (Constant) | 9.413 | 0.987 |
|  |  |  | Cq | -0.258 |  |
| **Lab3** | **Lab1** |  | (Constant) | 11.884 | 0.999 |
|  |  |  | Cq | -0.301 |  |
|  | **Lab2** |  | (Constant) | 11.884 | 0.999 |
|  |  |  | Cq | -0.301 |  |
|  | **Lab5** |  | (Constant) | 11.884 | 0.999 |
|  |  |  | Cq | -0.301 |  |
|  | **Lab3** |  | (Constant) | 11.884 | 0.999 |
|  |  |  | Cq | -0.301 |  |
|  | **Lab4** |  | (Constant) | 11.884 | 0.999 |
|  |  |  | Cq | -0.301 |  |
| **Lab4** | **Lab1** |  | (Constant) | 10.667 | 0.989 |
|  |  |  | Cq | -0.265 |  |
|  | **Lab2** |  | (Constant) | 11.077 | 0.999 |
|  |  |  | Cq | -0.275 |  |
|  | **Lab5** |  | (Constant) | 10.525 | 0.999 |
|  |  |  | Cq | -0.260 |  |
|  | **Lab3** |  | (Constant) | 10.512 | 0.999 |
|  |  |  | Cq | -0.260 |  |
|  | **Lab4** |  | (Constant) | 12.332 | 0.983 |
|  |  |  | Cq | -0.308 |  |

| .998 |
| --- |
| 1.000 |
| 1.000 |
| .930 |
| .974 |
